# Smartphone-based dispatch of Community First Responders in the United Kingdom

**DOI:** 10.1186/s13049-021-00888-0

**Published:** 2021-05-21

**Authors:** Adam James Rae Watson

**Affiliations:** Medical Science Division, University of Oxford, Oxford, UK

Dear Editors,

I read with great interest the recent article by Metelmann et al. [1], which reports a number of consensus statements regarding the smartphone-based dispatch of Community First Responders in Europe. One wonders if the British model of first responding was discussed by the authors at their conference, as I suggest it closely matches their reported consensus of European best practice.

In recent months, the ambulance services of the United Kingdom have rolled out a common smartphone application for volunteer Community First Responders across the country. The National Mobilisation Application Lite is linked directly with ambulance control rooms, allowing for the real-time tracking and rapid dispatch of responders, as well as easy two-way communication some ambulance services even have dispatchers exclusively for their Community First Responders.

In the United Kingdom, it is not uncommon for a Community First Responders to be dispatched to multiple incidents per day, as they will ordinarily be activated for any nearby high priority incident, which might range from fitting patients to reports of a cardiac arrest. In some regions, volunteers even operate marked response vehicles in areas of expected high demand, where they can provide valuable support to ambulance services.

I note that no consensus was reached on the need to prepare Community First Responders for acute psychological stress. As volunteers with limited training and experience, Community First Responders are frequently the first to reach very unwell patients, so I would argue that this preparation is essential.

My thanks to the authors for their intriguing perspective of how it is done on the continent.

**Adam Watson**.

Community First Responder, South Central Ambulance Service.

Medical Student, University of Oxford | adam.watson@hertford.ox.ac.uk

## Data Availability

Data sharing not applicable to this article as no datasets were generated or analysed during the current study.

## References

[1] Metelmann C, Metelmann B, Kohnen D, et al.Smartphone-based dispatch of community first responders to out-of-hospital cardiac arrest - statements from an international consensus conference. Scand J Trauma Resusc Emerg Med.2021;29:29. 10.1186/s13049-021-00841-1 PMC7852085 33526058

